# Supplementary material for: Hybrid Models and Biological Model Reduction with PyDSTool
Source: PLoS Comput Biol. 2012 Aug 9;8(8):e1002628. doi: 10.1371/journal.pcbi.1002628 (PMC3415397; doi:10.1371/journal.pcbi.1002628)
Supplement: Text S4 — Complete source code for the PyDSTool package (version 0.88.120504). Includes API documentation and help files linking to web pages. This file is identical to the current public release on Sourceforge.net. (ZIP) [file pcbi.1002628.s004.zip › PyDSTool/html/PyDSTool.matplotlib_import-pysrc.html]

xml version="1.0" encoding="ascii"?


PyDSTool.matplotlib\_import


| Home | Trees | Indices | Help | PyDSTool |
| --- | --- | --- | --- | --- |

Package PyDSTool ::
Module matplotlib\_import

[hide private]

[frames] | no frames]

# Source Code for Module PyDSTool.matplotlib\_import

```
 1  """
 
 2      Plotting imports for PyDSTool, from Matplotlib.
 
 3  
 
 4      Robert Clewley, March 2006.
 
 5  """ 
 6  
 
 7  # --------------------------------------------------------------------------
 
 8  # VERSION HISTORY
 
 9  #
 
10  # v1.1, August 2006
 
11  #   Added save_fig function to save figure in multiple formats.
 
12  #
 
13  # --------------------------------------------------------------------------
 
14  
 
15  __all__ = ['plot', 'pylab', 'save_fig'] 
16  
 
17  from numpy import Inf, NaN, isfinite, int, int8, int16, int32, int64, float, float32, float64 
18  import matplotlib 
19  ver = matplotlib.__version__.split(".") 
20  try: 
21      if int(ver[0]) == 0 and int(ver[1]) < 65: 
22          import matplotlib.matlab as pylab 
23      else: 
24          import matplotlib.pylab as pylab 
25  except RuntimeError, err: 
26      if str(err) == 'could not open display': 
27          failed=True 
28      else: 
29          raise 
30  else: 
31      failed=False 
32  
 
33  if failed: 
34      # Dummy plot overrides for PyDSTool when matplotlib fails to import
 
35  
 


36 -    def plot(*args, **kw):


37          print "Warning: plot does not work!"

38  
 


39 -    def save_fig(fignum, fname, formats=[]):


40          print "Warning: plot does not work!"

41  
 
42      print "Warning: matplotlib failed to import properly and so is not" 
43      print "  providing a graphing interface" 
44      pylab = None   # will cause an error if someone tries to access in order to plot 
45  else: 
46      import os 
47      from Trajectory import Trajectory 
48      from common import _num_types 
49  
 
50      # Convenient shorthand to permit singleton numeric types and Trajectories
 
51      # in the plot arguments without first converting them to lists or arrays.
 


52 -    def plot(*args, **kw):


53          new_args = list(args) 
54          if isinstance(args[0], _num_types): 
55              new_args[0] = [args[0]] 
56          elif isinstance(args[0], Trajectory): 
57              try: 
58                  new_args[0] = args[0].sample() 
59              except: 
60                  raise RuntimeError("Could not sample trajectory with default "
 
61                                     "options for plotting") 
62          if len(args) > 1: 
63              if isinstance(args[1], _num_types): 
64                  new_args[1] = [args[1]] 
65              elif isinstance(args[1], Trajectory): 
66                  try: 
67                      new_args[1] = args[1].sample() 
68                  except: 
69                      raise RuntimeError("Could not sample trajectory with "
 
70                                         "default options for plotting") 
71          return pylab.plot(*tuple(new_args), **kw)

72  
 


73 -    def save_fig(fignum, fname, formats=['png','svg','eps']):


74          """Save figure fignum to multiple files with different formats
 
75          and extensions given by the formats argument.
 
76          These are platform-dependent and are specific to matplotlib's support.
 
77          """ 
78          for f in formats: 
79              pylab.figure(fignum).savefig(fname+'.'+f)

80
```

  


| Home | Trees | Indices | Help | PyDSTool |
| --- | --- | --- | --- | --- |

|  |  |
| --- | --- |
| Generated by Epydoc 3.0.1 on Fri May 4 15:24:14 2012 | http://epydoc.sourceforge.net |
